# Supplementary material for: Personalized Prediction of Patient Radiation Exposure for Therapy of Urolithiasis: An Application and Comparison of Six Machine Learning Algorithms
Source: J Pers Med. 2023 Apr 7;13(4):643. doi: 10.3390/jpm13040643 (PMC10146849; doi:10.3390/jpm13040643)
Supplement: Supplementary file 1 [file jpm-13-00643-s001.zip › jpm-2305701-supplementary.pdf]

Supplementary Materials

Table S1. An overview about pre-processing, model training, split of data into training, validation and test sample, training stop criteria, selection of thresholds for the reject option and final test of the model.

	Description		Remarks
Encoding and input normalization	Input of the machine learing models were normalized so that each input has mean 0 and variance 1.	Categorical variables were encoded using one hot encoding	-
Description of training, validation and test samples	Full sample (*n* = 827) was randomly split into a training sample (*n* = 527) and test sample (*n* = 249). 10-fold cross-validation was used for model training using *n* = 57 of training data as validation set each. No data of the test sample was used for model training.	Two thresholds for the reject option were selected to maximize negative and positive predictive values and to minimize number of unpredicted cases.	The selected thresholds were evaluated in the training sample and also evaluated in the independent and randomly selected test sample (*n* = 249, Table 3)
Model training	Adaptive moment estimation were used as stochastic gradient descent method.	-	-
Training stopping criteria	Based on the network loss with a relative change of loss	Maximal training rounds ≤ 500 rounds	To mitigate overfitting, early stopping approach and 10-fold cross-validation were used. L2-regularization techniques were also used.
Model performances	Negative and positive predictive values were computed based on 10-fold cross-validation in all models (Table 2).		
Final test	Trained neural network models with two thresholds allowing the reject option were independently tested by evaluating negative, positive predictive power and percentage of unclassified subjects in the training and test sample. Results demonstrate that no overfitting occurred (Table 3)		

Table S2: Overview of model performances with various combinations of cut-offs for the neural network model.

		Training and validation sample			Test sample			
**Lower cut-off c1**	**Upper cut-off c2**	**Negative predictive value (NPV) (%)**	**Positive predictive value (PPV) (%)**	**Percentage of unpredicted cases (PUC) (%)**	**Negative predictive value (%)**	**Positive predictive value (%)**	**Percentage of unpredicted cases (PUC) (%)**	**Remarks**
0.5	0.5	487/578 84%	n.d.	0%	223/249 94%	n.d.	0%	This corresponds to a model without using a reject option.
0.1	0.5	487/578 84%	n.d.	0%	223/249 94%	n.d.	0%	NPV remained stable, PPV did not increase. PUC still remains 0%.
0.05	0.5	420/434 97%	n.d.	25%	178/188 95%	n.d.	25%	NPV and PUC considerably increased, PPV still not defined.
0.05	0.09	420/434 97%	41/62 66%	14%	178/188 95%	11/32 34%	12%	NPV stable, PPV increased, but was too low for application in clinical practice.
0.072	0.5	445/472 94%	n.d.	106/578 18%	186/198 94%	n.d.	51/249 20%	Final decision about the cut-offs and PUC was done after intensive discussions with the medical experts about their suggestions concerning NPV and PPV.

NPV and PUC were 94% and 19%, respectively after application of the reject option in the total sample (this corresponds to the model with reject option, Table 3). The cost for this improvement is that 19% of all patients in the total sample did not receive a prediction. Both selected cut-offs c1 and c2 were applied in a test sample to check whether results remain stable (Table 3).

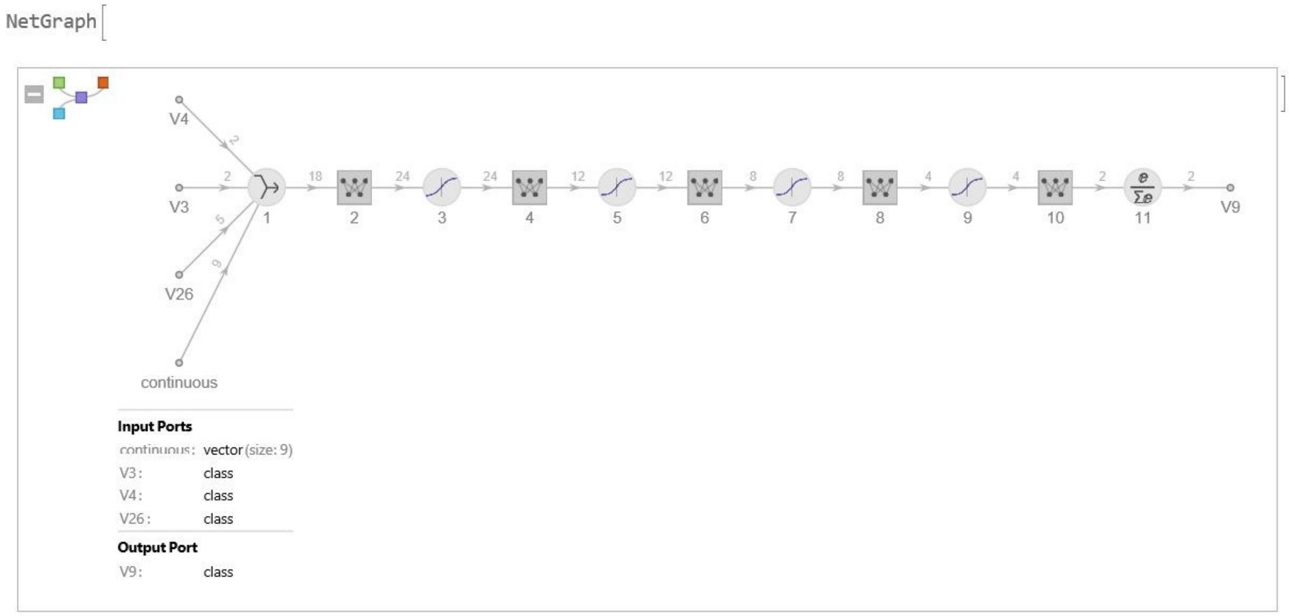

Figure S1: An illustration of the network architecture with all layers and activation functions to predict radiation exposure. Layers and activation functions are enumerated in the figure (1–11): (1) Catenation layer: Input vector consists of 3 discrete and 9 continuously distributed variables which were catenated by a catenate layer; (2) Linear layer, i.e. a real matrix with dimensions 24×18; (3) Hyperbolic tangent was used activation function; (4) Linear layer with dimensions 12×24; (5) Logistic sigmoid as activation function; (6) Linear layer with dimension 8×12; (7) Logistic sigmoid as activation function; (8) Linear layer with dimensions 4×8; (9) Logistic sigmoid; (10) Linear layer with dimensions 2×4; (11) Softmax layer.
